# Supplementary material for: Controlling for Variable Transposition Rate with an Age-Adjusted Site Frequency Spectrum
Source: Genome Biol Evol. 2022 Feb 1;14(2):evac016. doi: 10.1093/gbe/evac016 (PMC8872973; doi:10.1093/gbe/evac016)
Supplement: evac016_Supplementary_Data [file evac016_supplementary_data.zip › Horvath et al. Supplementary Material.docx]

**Supplementary Material**

**Controlling for variable transposition rate with an age-adjusted site frequency spectrum**

Robert Horvath^1*^, Mitra Menon^1,2^, Michelle Stitzer^3^, Jeffrey Ross-Ibarra^1,2,4*^

^1^Department of Evolution and Ecology, University of California, One Shields Avenue, Davis, CA, USA

^2^Center for Population Biology, University of California, One Shields Avenue, Davis, CA, USA

^3^Institute for Genomic Diversity and Department of Molecular Biology and Genetics, Cornell University, Ithaca, NY, 14850, USA

^4^Genome Center, University of California, One Shields Avenue, Davis, CA, USA

*Corresponding author:

Robert Horvath: [robert.horvath@bluewin.ch](mailto:robert.horvath@bluewin.ch)

Jeffrey Ross-Ibarra: [rossibarra@ucdavis.edu](mailto:rossibarra@ucdavis.edu)

**Supplementary Tables**

**Table S1**. Percent of runs which showed a significant negative correlation between Δ frequency and age at different timepoints (in generations: G) in our simulations (*p*-value < 0.05). The third column indicates the observations made after the burn in phase at generation 0 (G 0), which corresponds to ideal conditions (constant demography and transposition rate).

| Model | 4**N_e_***s* | G 0 | G 50 | G 250 | G 300 | G 500 | G 750 | G 1250 | G 2000 | G 5000 |
| --- | --- | --- | --- | --- | --- | --- | --- | --- | --- | --- |
| Bottleneck | |  |  |  |  |  |  |  |  |  |
|  | 0 | 9% | 4% | 8% | 17% | 10% | 11% | 12% | 9% | 9% |
|  | -0.2 | 26% | 7% | 6% | 16% | 13% | 16% | 20% | 28% | 32% |
|  | -2 | 96% | 35% | 17% | 25% | 48% | 82% | 82% | 88% | 92% |
|  | -10 | 97% | 61% | 31% | 69% | 94% | 97% | 98% | 98% | 99% |
|  | -20 | 99% | 65% | 47% | 89% | 95% | 99% | 96% | 97% | 99% |
| TE burst | |  |  |  |  |  |  |  |  |  |
|  | 0 | 8% | 8% | 7% | 6% | 5% | 10% | 4% | 5% | 4% |
|  | -0.2 | 29% | 10% | 27% | 15% | 28% | 35% | 44% | 45% | 48% |
|  | -2 | 95% | 47% | 87% | 99% | 100% | 100% | 99% | 100% | 100% |
|  | -10 | 98% | 90% | 100% | 100% | 100% | 100% | 100% | 100% | 100% |
|  | -20 | 97% | 99% | 100% | 100% | 100% | 100% | 100% | 100% | 100% |
| Bottleneck and TE burst | | |  |  |  |  |  |  |  |  |
|  | 0 | 10% | 4% | 10% | 7% | 14% | 11% | 10% | 3% | 8% |
|  | -0.2 | 23% | 10% | 5% | 9% | 26% | 28% | 27% | 38% | 44% |
|  | -2 | 95% | 42% | 13% | 60% | 93% | 96% | 99% | 100% | 100% |
|  | -10 | 98% | 47% | 67% | 100% | 100% | 100% | 100% | 99% | 100% |
|  | -20 | 99% | 66% | 91% | 100% | 100% | 100% | 100% | 100% | 100% |

**Table S2**. Range (minimum – maximum) of segregating TE insertions in the population at different timepoints (in generations: G) in our simulations.

| Model | 4**N_e_***s* | G 0 | G 50 | G 250 | G 300 | G 500 | G 750 | G 1250 | G 2000 | G 5000 |
| --- | --- | --- | --- | --- | --- | --- | --- | --- | --- | --- |
| Bottleneck | |  |  |  |  |  |  |  |  |  |
|  | 0 | 9 238-  9 853 | 2 518-  2 830 | 856-  1 018 | 4 937-  5 413 | 6 839-  7 407 | 7 713-  8 298 | 8 684-  9 445 | 9 590-  10 403 | 11 773-  12 643 |
|  | -0.2 | 8 724-  9 447 | 2 388-  2 753 | 793-  985 | 4 748-  5 218 | 6 569-  7 084 | 7 295-  7 951 | 8 202-  9 008 | 9 025-  9 871 | 10 662-  11 716 |
|  | -2 | 6 183-  6 654 | 1 402-  1 665 | 563-  728 | 3 567-  3 945 | 4 854-  5 272 | 5 335-  5 847 | 5 809-  6 322 | 6 086-  6 624 | 5 935-  6 523 |
|  | -10 | 3 656-  4 035 | 489-  628 | 346-  449 | 2 746-  3 052 | 3 453-  3 812 | 3 564-  3 932 | 3 420-  3 836 | 3 349-  3 704 | 2 893-  3 213 |
|  | -20 | 2 969-  3 349 | 327-  455 | 283-  396 | 2 551-  2 829 | 2 945-  3 277 | 2 875-  3 239 | 2 884-  3 156 | 2 746-  3 040 | 2 365-  2 571 |
| TE burst | |  |  |  |  |  |  |  |  |  |
|  | 0 | 8 987-  9 846 | 53 024-  57 161 | 87 204-  93 648 | 42 606-  45 500 | 30 185-  32 445 | 28 242-  30 116 | 28 449-  30 988 | 30 717-  33 119 | 46 721-  50 537 |
|  | -0.2 | 8 604-  9 410 | 51 400-  53 960 | 83 653-  88 956 | 40 684-  43 422 | 28 466-  30 442 | 26 504-  28 315 | 26 698-  28 499 | 28 124-  30 239 | 40 560-  44 221 |
|  | -2 | 6 191-  6 664 | 39 188-  41 480 | 62 226-  65 809 | 29 189-  30 879 | 19 538-  20 764 | 17 085-  18 216 | 15 804-  16 899 | 15 322-  16 555 | 15 748-  17 233 |
|  | -10 | 3 656-  4 004 | 29 406-  31 292 | 39 631-  42 590 | 16 049-  17 397 | 9 451-  10 204 | 7 915-  8 533 | 7 172-  7 711 | 6 818-  7 332 | 5 894-  6 357 |
|  | -20 | 3 030-  3 317 | 26 411-  28 279 | 31 355-  33 494 | 10 588-  11 675 | 6 477-  7 034 | 5 883-  6 443 | 5 694-  6 226 | 5 485-  5 992 | 4 678-  5 225 |
| Bottleneck and TE burst | | |  |  |  |  |  |  |  |  |
|  | 0 | 9 209-  9 961 | 6 851-  7 576 | 8 568-  9 657 | 17 570-  19 128 | 20 459-  22 034 | 22 242-  23 953 | 25 377-  27 179 | 29 474-  31 339 | 47 012-  50 646 |
|  | -0.2 | 8 718-  9 416 | 6 728-  7 369 | 8 216-  9 283 | 16 866-  18 206 | 19 552-  21 129 | 21 427-  23 072 | 24 048-  25 673 | 27 444-  29 238 | 41 693-  44 787 |
|  | -2 | 6 206-  6 714 | 4 743-  5 311 | 6 215-  7 047 | 12 709-  13 694 | 14 322-  15 692 | 15 251-  16 404 | 15 913-  17 110 | 16 363-  17 656 | 17 650-  19 046 |
|  | -10 | 3 653-  3 998 | 2 982-  3 458 | 4 207-  4 954 | 8 690-  9 737 | 8 959-  9 739 | 8 491-  9 120 | 7 829-  8 439 | 7 358-  8 000 | 6 333-  6 923 |
|  | -20 | 3 012-  3 320 | 2 606-  3 134 | 3 376-  4 013 | 7 222-  7 875 | 6 769-  7 254 | 6 359-  6 822 | 5 994-  6 517 | 5 766-  6 361 | 4 960-  5 479 |

Table S3. Percent of runs which showed a significant negative correlation between Δ frequency and age (p-value < 0.05) after sampling 50, 20 and 10 individuals as well as limiting the number of segregating TE insertions to 2 000, 1 000, 500 and 100. An age-adjusted SFS analysis was performed in all models with a scaled selection coefficient of -10 at generation 250. The numbers in the brackets indicate the range (minimum – maximum) of segregating TE insertions for analyses where the number of sampled individuals was limited. Note that in the bottleneck model even when all individuals were sampled the total number of segregating TE insertions was less than 500.

| Model |  | Percent of runs |
| --- | --- | --- |
| Bottleneck | |  |
|  | 50 individuals sampled (346-449) | 31% |
|  | 20 individuals sampled (274-370) | 27% |
|  | 10 individuals sampled (227-295) | 23% |
|  | 2 000 segregating TEs | Na |
|  | 1 000 segregating TEs | Na |
|  | 500 segregating TEs | Na |
|  | 100 segregating TEs | 29% |
| TE burst | |  |
|  | 50 individuals sampled (18 840-20 676) | 100% |
|  | 20 individuals sampled (12 426-13 687) | 100% |
|  | 10 individuals sampled (8 296-9 091) | 100% |
|  | 2 000 segregating TEs | 83% |
|  | 1 000 segregating TEs | 68% |
|  | 500 segregating TEs | 56% |
|  | 100 segregating TEs | 27% |
| Bottleneck and TE burst | |  |
|  | 50 individuals sampled (4 207-4 954) | 67% |
|  | 20 individuals sampled (3 143-3 809) | 65% |
|  | 10 individuals sampled (2 687-3 185) | 53% |
|  | 2 000 segregating TEs | 61% |
|  | 1 000 segregating TEs | 33% |
|  | 500 segregating TEs | 36% |
|  | 100 segregating TEs | 13% |

**Supplementary Figures**

**Figure S1.** Expected (Kimura and Ohta 1973; Maruyama 1974) and observed mean age distribution of TEs at a specific frequency based on 100 runs after the burn in phase (Generation 0). Plot A_1_ – A_4_: bottleneck model and plot B_1_ – B_4_: TE burst model. The subscript 1 to 4 indicates the strength of selection with the scaled selection coefficient corresponding to 0, -2, -10, -20, respectively.


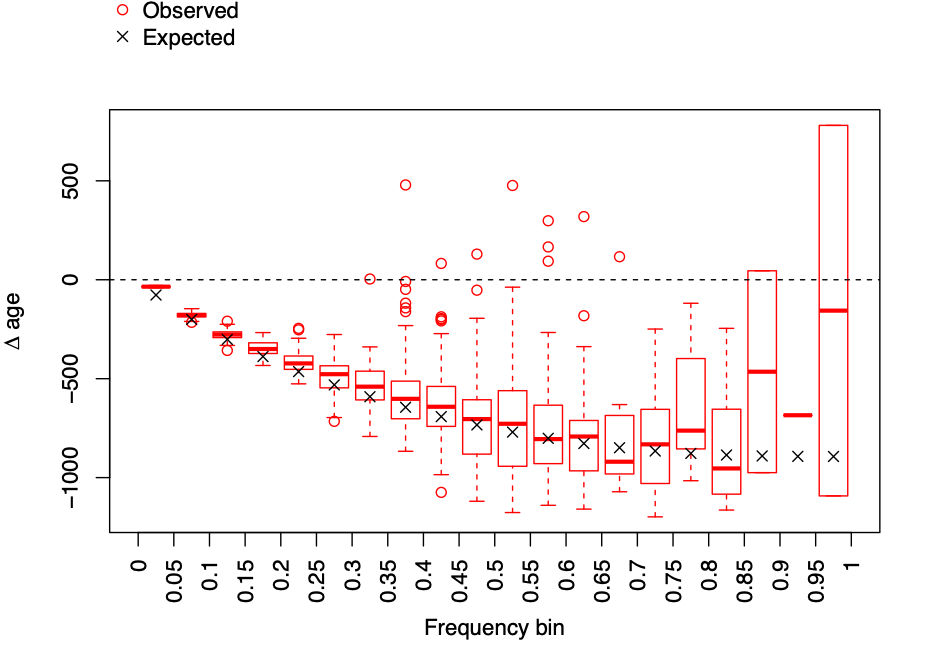


**Figure S2**. Difference in age between TEs and SNPs (Δ age: mean TE age – mean SNP age) at a specific frequency caused by negative selection. Observed Δ age between TEs under selection (scaled selection coefficient -10) and neutrally evolving SNPs are shown in red and expected Δ age (mean allele age predicted by Maruyama (1974) - mean allele age predicted by Kimura and Ohta (1973)) are shown in black.


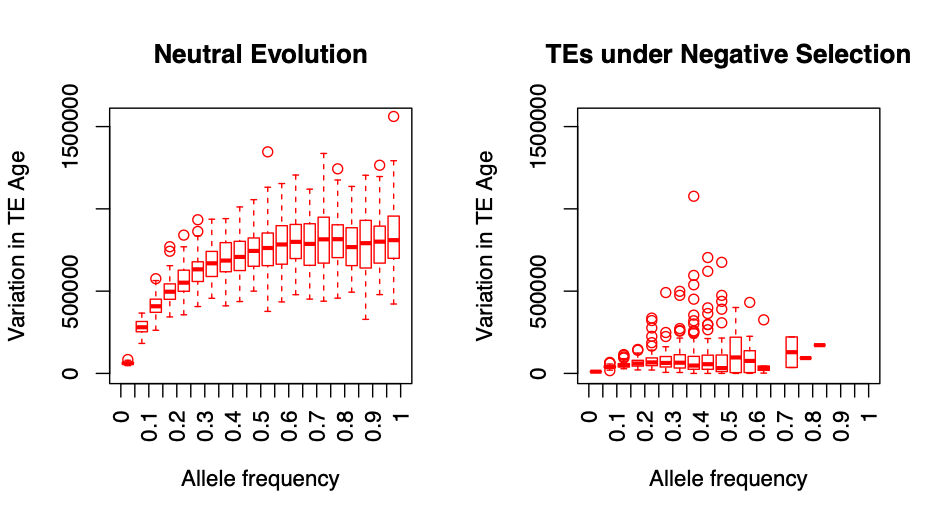


Figure S3. Variation in the TE ages at split by frequencies for neutrally evolving TEs (left panel) and for TEs under negative selection (4Nes = -10; right panel) after the burn-in phase.


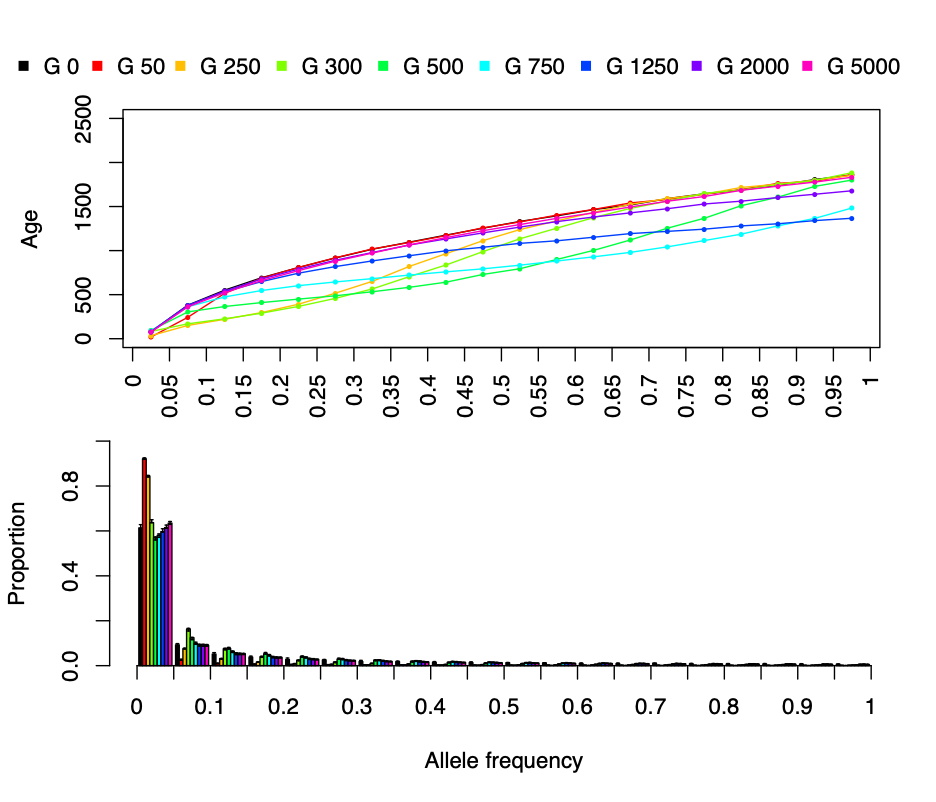


**Figure S4.** Mean age distribution and proportion of TEs at a given frequency in our TE burst model at the end of the burn in phase (G 0), generation 50 (G 50), 250 (G 250), 300 (G 300), 500 (G 500), 750 (G 750), 1250 (G 1250), 2000 (G 2000) and 5000 (G 5000). Top panel: mean TE age distribution. Bottom panel: TE site frequency spectrum (SFS).

**Figure S5**. Age binned Δ frequency (mean TE frequency – mean SNP frequency) distributions observed in the different models. A. Observed Δ frequency between neutrally evolving TEs and SNPs under a bottleneck model. B. Observed Δ frequency between negatively selected TEs (4*N_e_s* = -10) and neutrally evolving SNPs under a bottleneck model. C. Observed Δ frequency between neutrally evolving TEs and SNPs under a TE burst model. D. Observed Δ frequency between negatively selected TEs (4*N_e_s* = -10) and neutrally evolving SNPs under a TE burst model. The four rows represent samples from generations 300, 500, 2000 and 5000 respectively.

1. B.


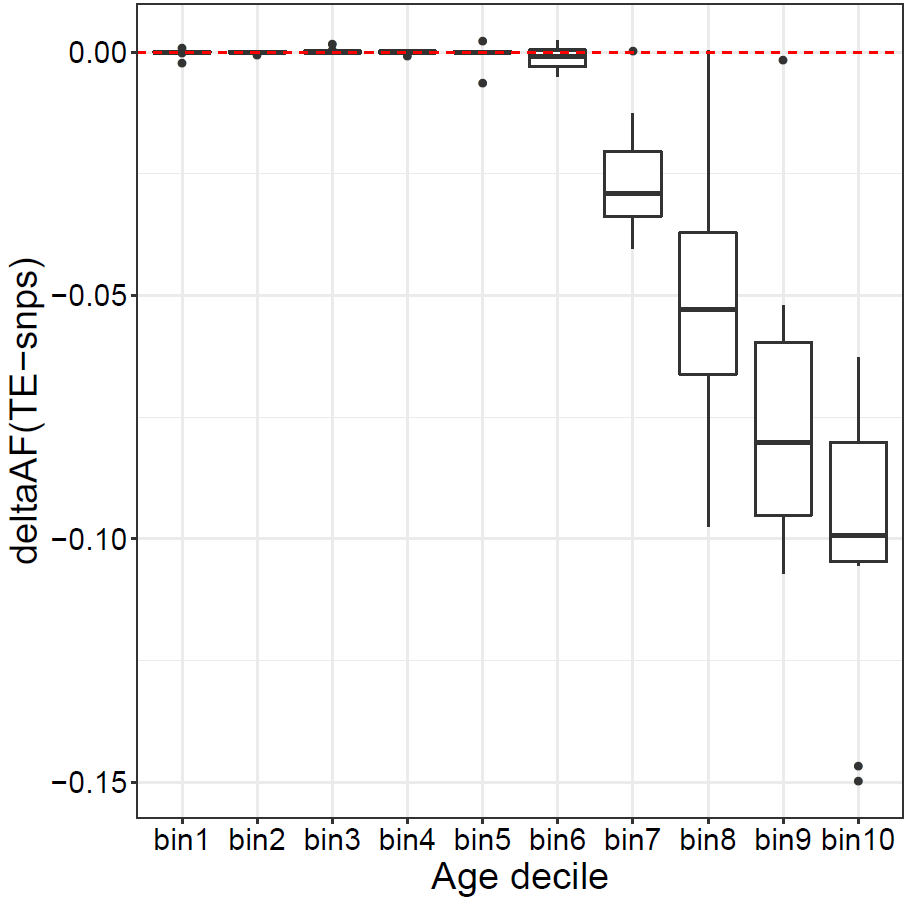

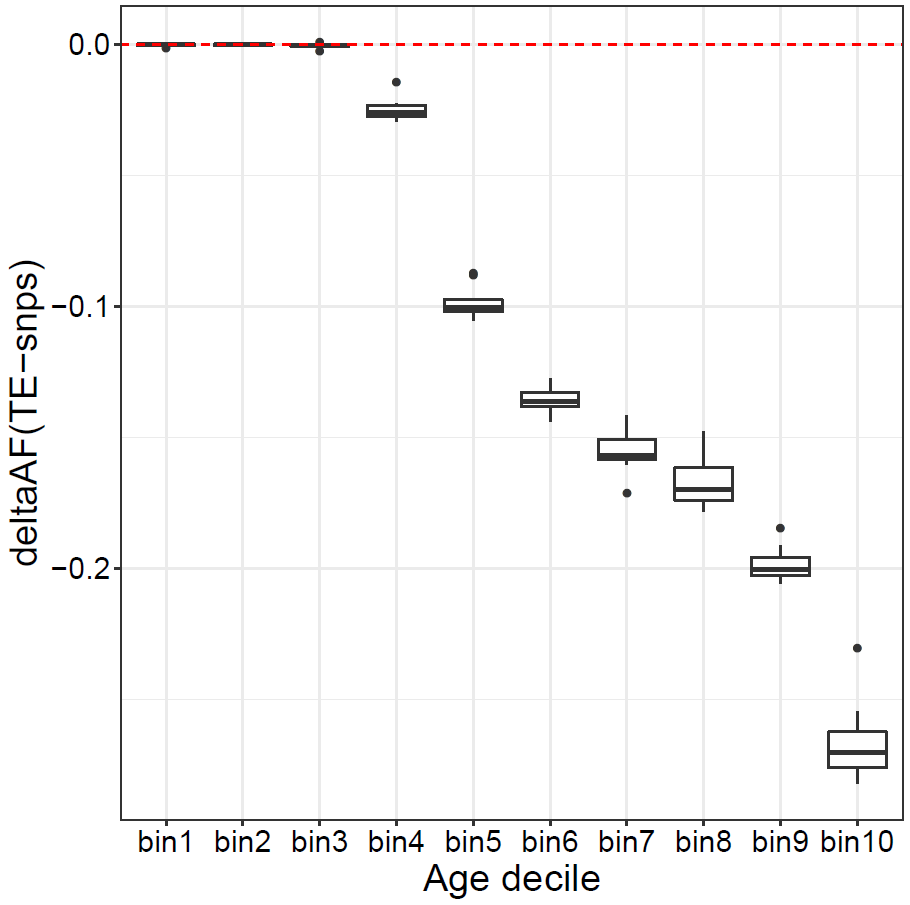


**Figure S6.** Age binned Δ frequency (mean TE frequency – mean SNP frequency) distributions after conditioning on empirical age estimates obtained from GEVA under A) a bottleneck model with negatively selected TEs (4*N_e_s* = -10) and under B) a TE burst model with negatively selected TEs (4*N_e_s* = -10). Note that singletons were not included in this analysis because of high inaccuracies in their age estimates.


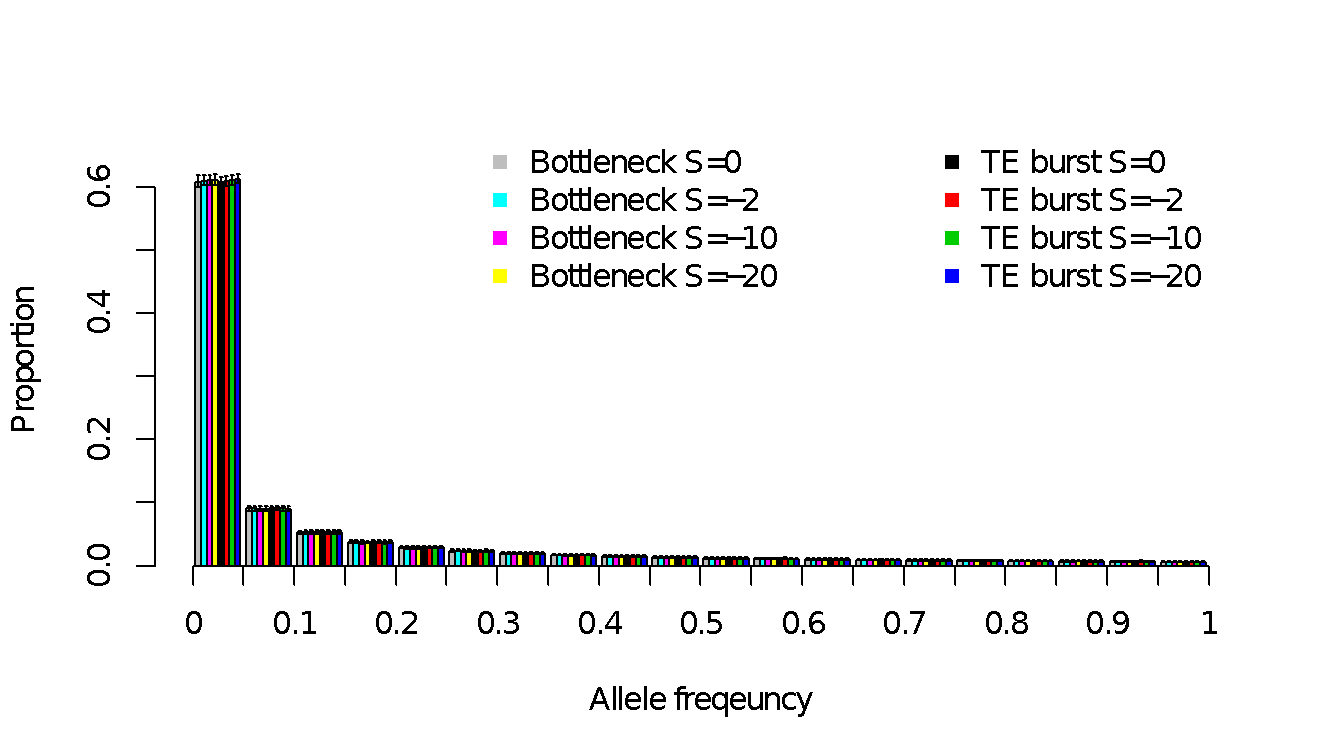


**Figure S7.** SNP SFS after the burn in phase in the different models.

**Supplementary References**

Kimura M, Ohta T. 1973. The age of a neutral mutant persisting in a finite population. *Genetics* 75(1): 199–212.

Maruyama T. 1974. The age of an allele in a finite population. *Genetical Research* 23(2): 137–143.
