# Supplementary material for: Controlling for Variable Transposition Rate with an Age-Adjusted Site Frequency Spectrum
Source: Genome Biol Evol. 2022 Feb 1;14(2):evac016. doi: 10.1093/gbe/evac016 (PMC8872973; doi:10.1093/gbe/evac016)
Supplement: evac016_Supplementary_Data [file evac016_supplementary_data.zip › Horvath et al. Supplementary Material.pdf]

Supplementary Material

Controlling for variable transposition rate with an age-adjusted site frequency spectrum

Robert Horvath[1*], Mitra Menon[1,2], Michelle Stitzer[3], Jeffrey Ross-Ibarra[1,2,4*]

[1]Department of Evolution and Ecology, University of California, One Shields Avenue, Davis, CA, USA

[2]Center for Population Biology, University of California, One Shields Avenue, Davis, CA, USA

[3]Institute for Genomic Diversity and Department of Molecular Biology and Genetics, Cornell University, Ithaca, NY, 14850, USA

[4]Genome Center, University of California, One Shields Avenue, Davis, CA, USA

*Corresponding author:
Robert Horvath: robert.horvath@bluewin.ch
Jeffrey Ross-Ibarra: rossibarra@ucdavis.edu

Supplementary Tables

Table S1. Percent of runs which showed a significant negative correlation between Δ frequency and age at different timepoints (in generations: G) in our simulations (*p*-value < 0.05). The third column indicates the observations made after the burn in phase at generation 0 (G 0), which corresponds to ideal conditions (constant demography and transposition rate).

Model	$4*N_e*s$	G 0	G 50	G 250	G 300	G 500	G 750	G 1250	G 2000	G 5000
Bottleneck										
	0	9%	4%	8%	17%	10%	11%	12%	9%	9%
	-0.2	26%	7%	6%	16%	13%	16%	20%	28%	32%
	-2	96%	35%	17%	25%	48%	82%	82%	88%	92%
	-10	97%	61%	31%	69%	94%	97%	98%	98%	99%
	-20	99%	65%	47%	89%	95%	99%	96%	97%	99%
TE burst										
	0	8%	8%	7%	6%	5%	10%	4%	5%	4%
	-0.2	29%	10%	27%	15%	28%	35%	44%	45%	48%
	-2	95%	47%	87%	99%	100%	100%	99%	100%	100%
	-10	98%	90%	100%	100%	100%	100%	100%	100%	100%
	-20	97%	99%	100%	100%	100%	100%	100%	100%	100%
Bottleneck and TE burst										
	0	10%	4%	10%	7%	14%	11%	10%	3%	8%
	-0.2	23%	10%	5%	9%	26%	28%	27%	38%	44%
	-2	95%	42%	13%	60%	93%	96%	99%	100%	100%
	-10	98%	47%	67%	100%	100%	100%	100%	99%	100%
	-20	99%	66%	91%	100%	100%	100%	100%	100%	100%

Table S2. Range (minimum – maximum) of segregating TE insertions in the population at different timepoints (in generations: G) in our simulations.

Model	$4*N_e*s$	G 0	G 50	G 250	G 300	G 500	G 750	G 1250	G 2000	G 5000
Bottleneck										
	0	9 238-9 853	2 518-2 830	856-1 018	4 937-5 413	6 839-7 407	7 713-8 298	8 684-9 445	9 590-10 403	11 773-12 643
	-0.2	8 724-9 447	2 388-2 753	793-985	4 748-5 218	6 569-7 084	7 295-7 951	8 202-9 008	9 025-9 871	10 662-11 716
	-2	6 183-6 654	1 402-1 665	563-728	3 567-3 945	4 854-5 272	5 335-5 847	5 809-6 322	6 086-6 624	5 935-6 523
	-10	3 656-4 035	489-628	346-449	2 746-3 052	3 453-3 812	3 564-3 932	3 420-3 836	3 349-3 704	2 893-3 213
	-20	2 969-3 349	327-455	283-396	2 551-2 829	2 945-3 277	2 875-3 239	2 884-3 156	2 746-3 040	2 365-2 571
TE burst										
	0	8 987-9 846	53 024-57 161	87 204-93 648	42 606-45 500	30 185-32 445	28 242-30 116	28 449-30 988	30 717-33 119	46 721-50 537
	-0.2	8 604-9 410	51 400-53 960	83 653-88 956	40 684-43 422	28 466-30 442	26 504-28 315	26 698-28 499	28 124-30 239	40 560-44 221
	-2	6 191-6 664	39 188-41 480	62 226-65 809	29 189-30 879	19 538-20 764	17 085-18 216	15 804-16 899	15 322-16 555	15 748-17 233
	-10	3 656-4 004	29 406-31 292	39 631-42 590	16 049-17 397	9 451-10 204	7 915-8 533	7 172-7 711	6 818-7 332	5 894-6 357
	-20	3 030-3 317	26 411-28 279	31 355-33 494	10 588-11 675	6 477-7 034	5 883-6 443	5 694-6 226	5 485-5 992	4 678-5 225
Bottleneck and TE burst										
	0	9 209-9 961	6 851-7 576	8 568-9 657	17 570-19 128	20 459-22 034	22 242-23 953	25 377-27 179	29 474-31 339	47 012-50 646
	-0.2	8 718-9 416	6 728-7 369	8 216-9 283	16 866-18 206	19 552-21 129	21 427-23 072	24 048-25 673	27 444-29 238	41 693-44 787
	-2	6 206-6 714	4 743-5 311	6 215-7 047	12 709-13 694	14 322-15 692	15 251-16 404	15 913-17 110	16 363-17 656	17 650-19 046
	-10	3 653-3 998	2 982-3 458	4 207-4 954	8 690-9 737	8 959-9 739	8 491-9 120	7 829-8 439	7 358-8 000	6 333-6 923
	-20	3 012-3 320	2 606-3 134	3 376-4 013	7 222-7 875	6 769-7 254	6 359-6 822	5 994-6 517	5 766-6 361	4 960-5 479

Table S3. Percent of runs which showed a significant negative correlation between Δ frequency and age (p-value < 0.05) after sampling 50, 20 and 10 individuals as well as limiting the number of segregating TE insertions to 2 000, 1 000, 500 and 100. An age-adjusted SFS analysis was performed in all models with a scaled selection coefficient of -10 at generation 250. The numbers in the brackets indicate the range (minimum – maximum) of segregating TE insertions for analyses where the number of sampled individuals was limited. Note that in the bottleneck model even when all individuals were sampled the total number of segregating TE insertions was less than 500.

Model		Percent of runs
Bottleneck		
	50 individuals sampled (346-449)	31%
	20 individuals sampled (274-370)	27%
	10 individuals sampled (227-295)	23%
	2 000 segregating TEs	Na
	1 000 segregating TEs	Na
	500 segregating TEs	Na
	100 segregating TEs	29%
TE burst		
	50 individuals sampled (18 840-20 676)	100%
	20 individuals sampled (12 426-13 687)	100%
	10 individuals sampled (8 296-9 091)	100%
	2 000 segregating TEs	83%
	1 000 segregating TEs	68%
	500 segregating TEs	56%
	100 segregating TEs	27%
Bottleneck and TE burst		
	50 individuals sampled (4 207-4 954)	67%
	20 individuals sampled (3 143-3 809)	65%
	10 individuals sampled (2 687-3 185)	53%
	2 000 segregating TEs	61%
	1 000 segregating TEs	33%
	500 segregating TEs	36%
	100 segregating TEs	13%

Supplementary Figures

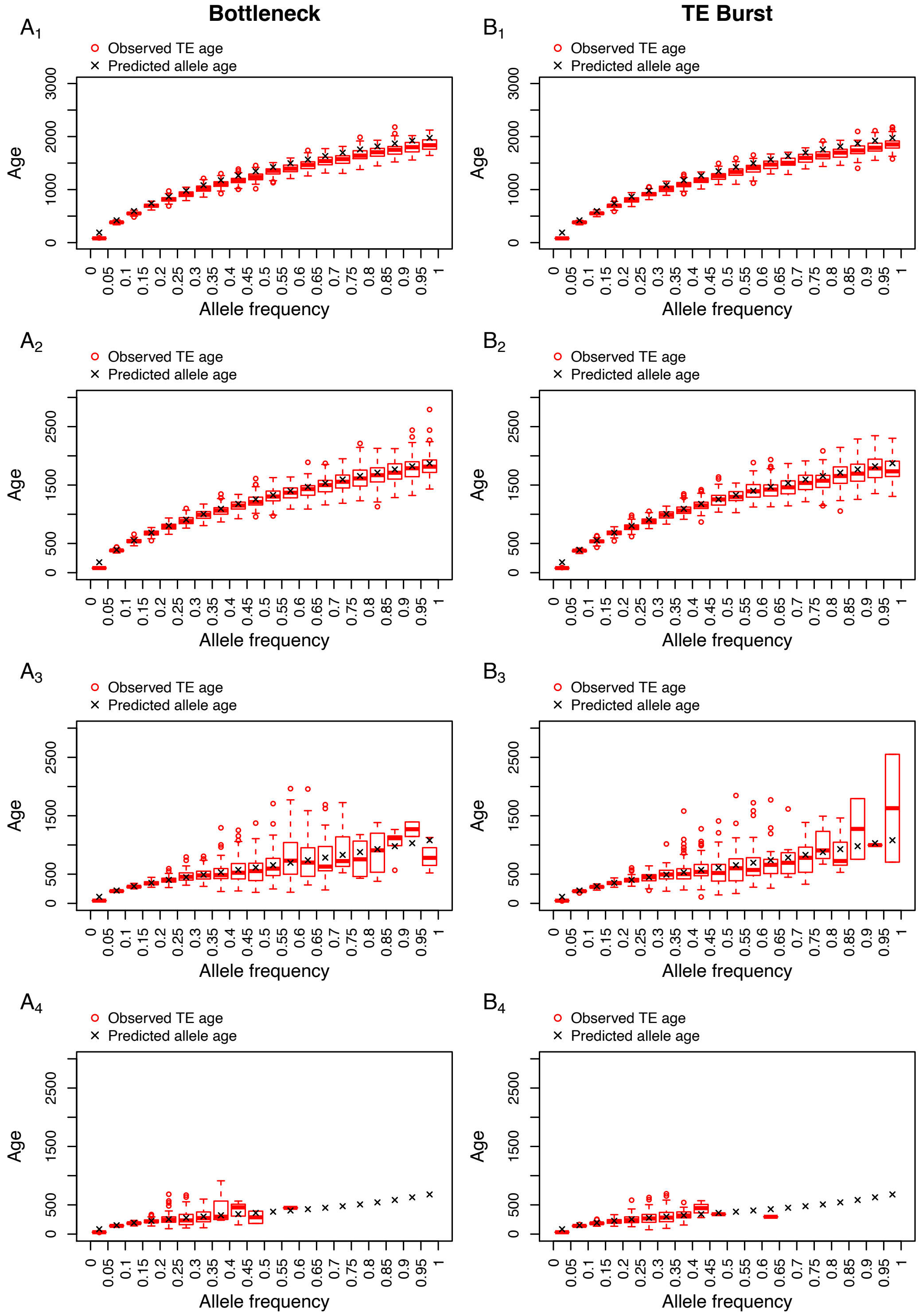

Figure S1. Expected (Kimura and Ohta 1973; Maruyama 1974) and observed mean age distribution of TEs at a specific frequency based on 100 runs after the burn in phase (Generation 0). Plot A_1 – A_4: bottleneck model and plot B_1 – B_4: TE burst model. The subscript 1 to 4 indicates the strength of selection with the scaled selection coefficient corresponding to 0, -2, -10, -20, respectively.

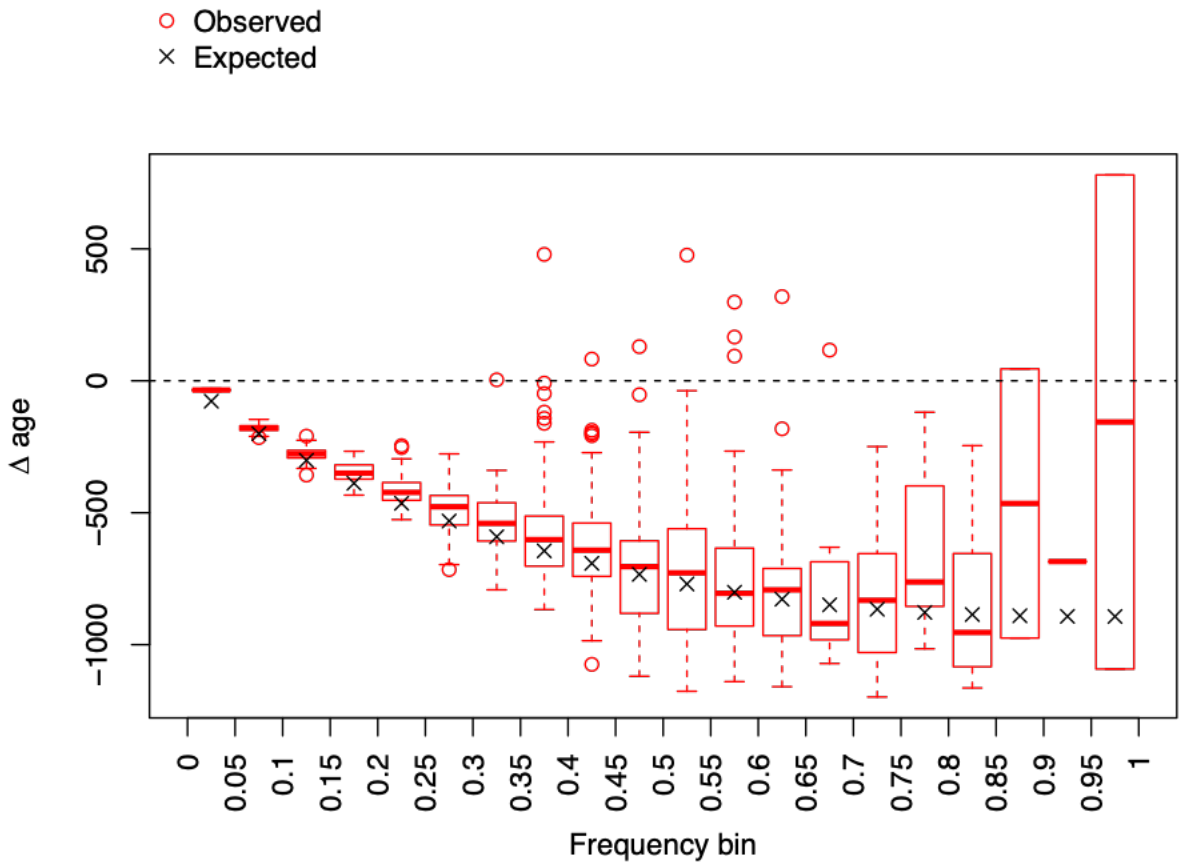

Figure S2. Difference in age between TEs and SNPs (Δ age: mean TE age – mean SNP age) at a specific frequency caused by negative selection. Observed Δ age between TEs under selection (scaled selection coefficient -10) and neutrally evolving SNPs are shown in red and expected Δ age (mean allele age predicted by Maruyama (1974) - mean allele age predicted by Kimura and Ohta (1973)) are shown in black.

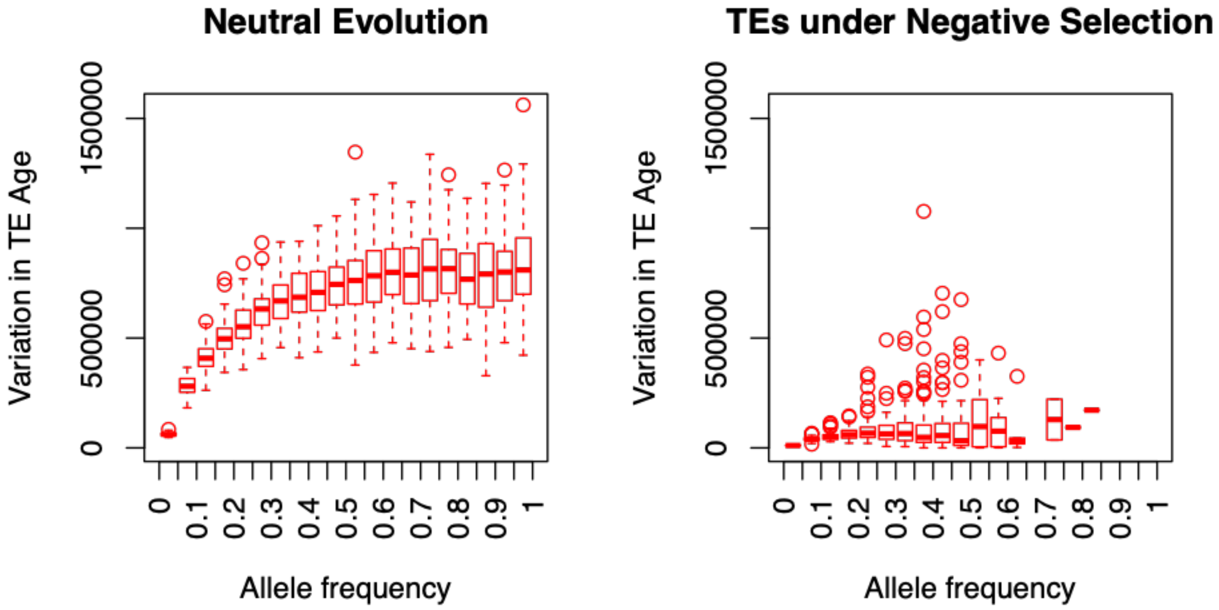

Figure S3. Variation in the TE ages at split by frequencies for neutrally evolving TEs (left panel) and for TEs under negative selection (4Nes = -10; right panel) after the burn-in phase.

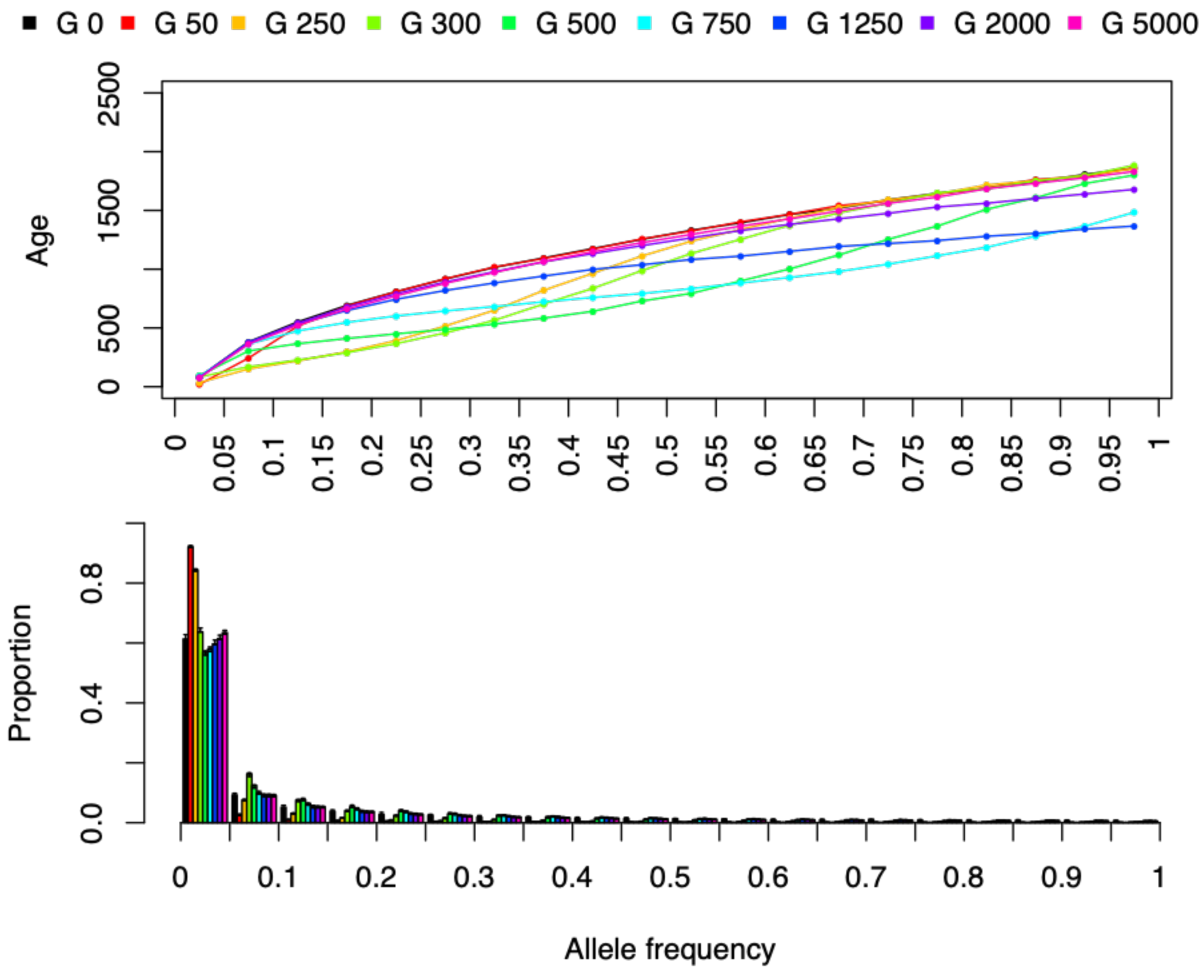

Figure S4. Mean age distribution and proportion of TEs at a given frequency in our TE burst model at the end of the burn in phase (G 0), generation 50 (G 50), 250 (G 250), 300 (G 300), 500 (G 500), 750 (G 750), 1250 (G 1250), 2000 (G 2000) and 5000 (G 5000). Top panel: mean TE age distribution. Bottom panel: TE site frequency spectrum (SFS).

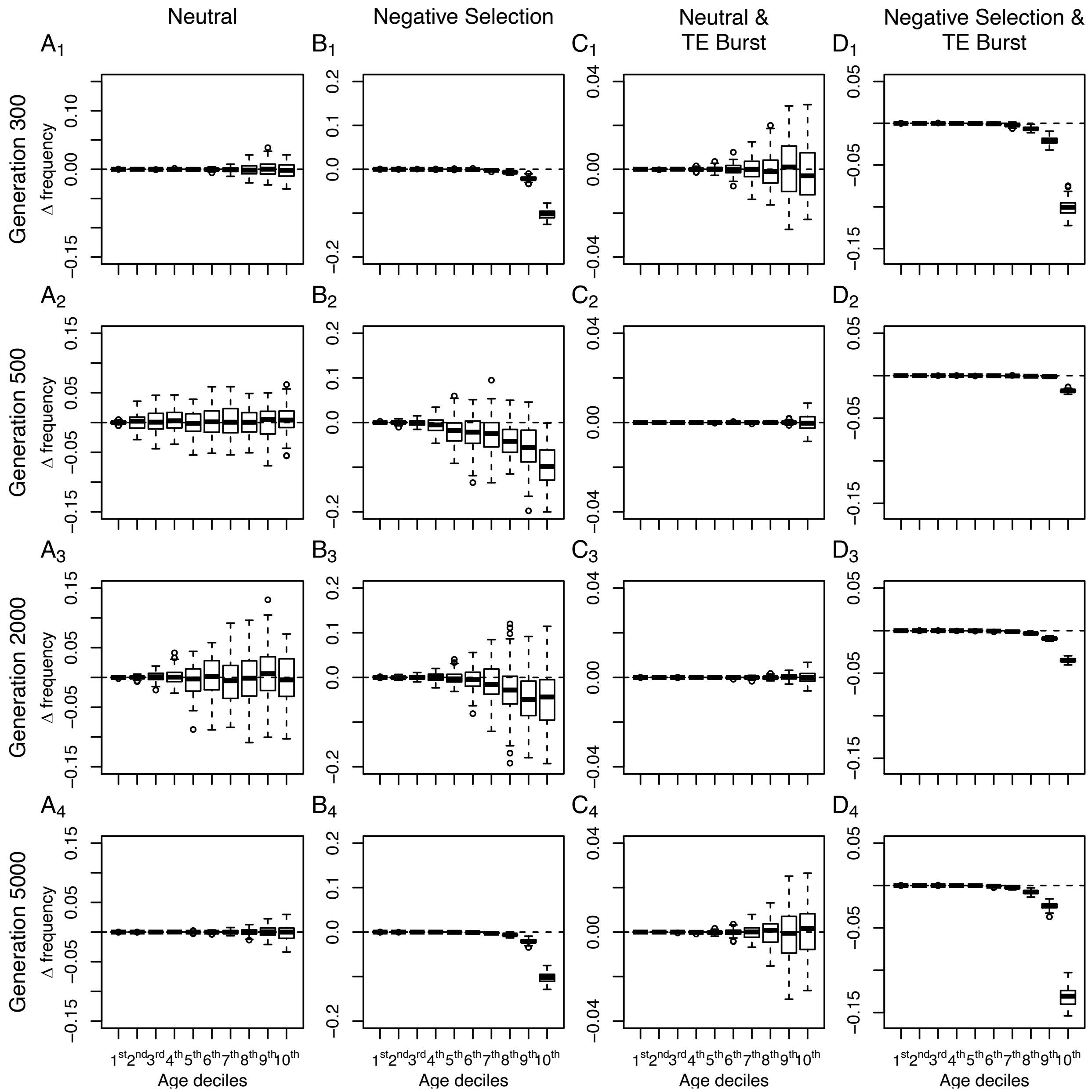

Figure S5. Age binned Δ frequency (mean TE frequency – mean SNP frequency) distributions observed in the different models. A. Observed Δ frequency between neutrally evolving TEs and SNPs under a bottleneck model. B. Observed Δ frequency between negatively selected TEs ($4N_es$ = -10) and neutrally evolving SNPs under a bottleneck model. C. Observed Δ frequency between neutrally evolving TEs and SNPs under a TE burst model. D. Observed Δ frequency between negatively selected TEs ($4N_es$ = -10) and neutrally evolving SNPs under a TE burst model. The four rows represent samples from generations 300, 500, 2000 and 5000 respectively.

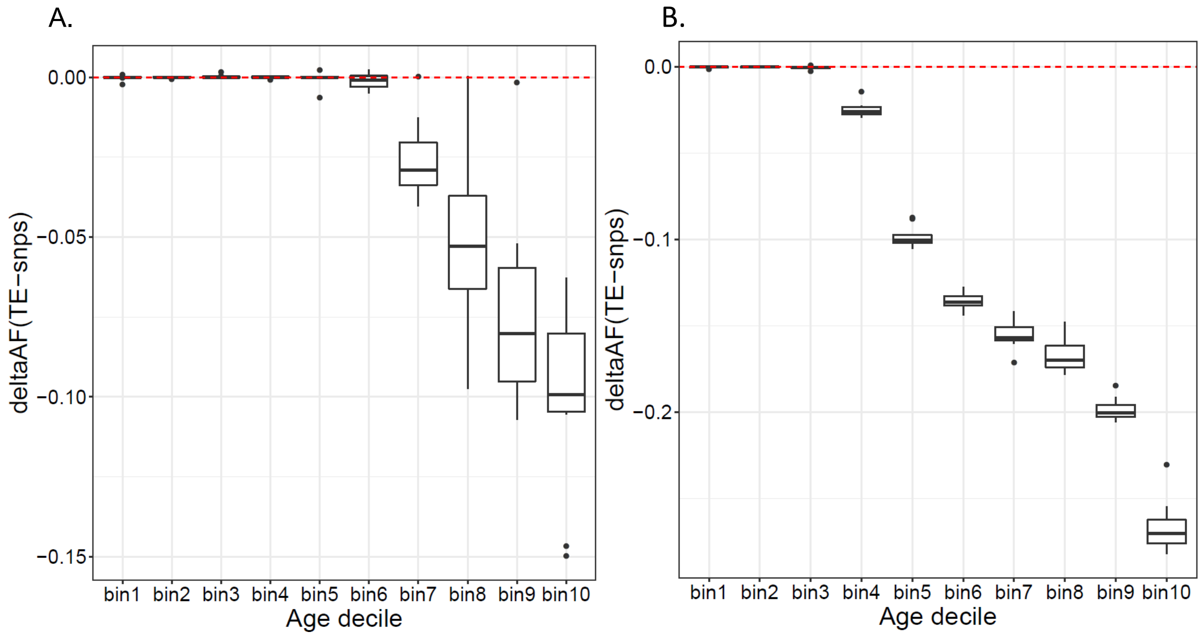

Figure S6. Age binned Δ frequency (mean TE frequency – mean SNP frequency) distributions after conditioning on empirical age estimates obtained from GEVA under A) a bottleneck model with negatively selected TEs ($4N_es$ = -10) and under B) a TE burst model with negatively selected TEs ($4N_es$ = -10). Note that singletons were not included in this analysis because of high inaccuracies in their age estimates.

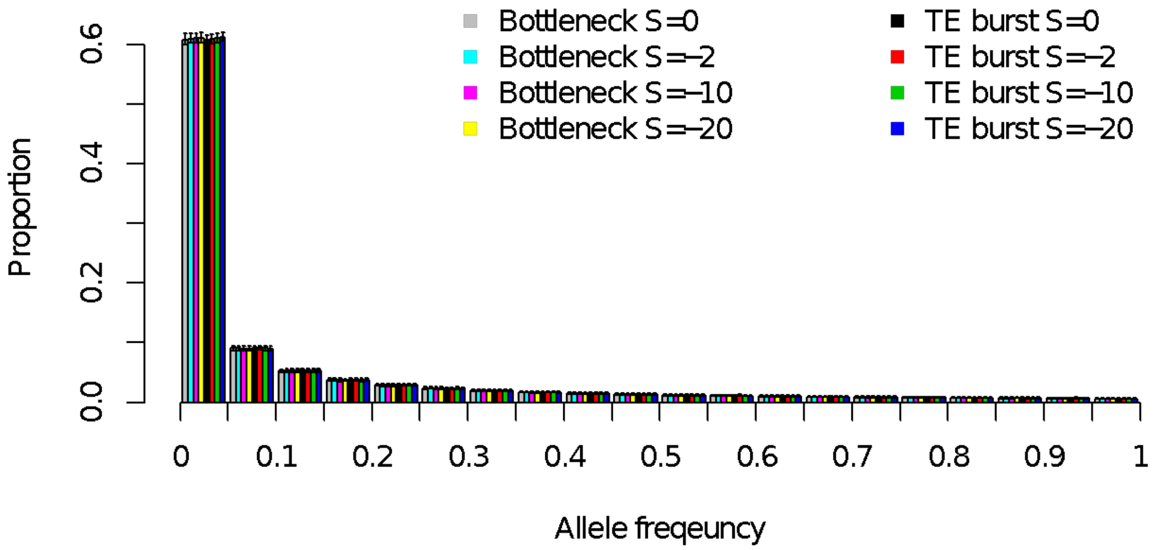

Figure S7. SNP SFS after the burn in phase in the different models.

Supplementary References

Kimura M, Ohta T. 1973. The age of a neutral mutant persisting in a finite population. *Genetics* 75(1): 199–212.

Maruyama T. 1974. The age of an allele in a finite population. *Genetical Research* 23(2): 137–143.
